# Perceptual–Cognitive Abilities and Reaction Performance in Female Volleyball Players: Implications for Training and Player Development

**DOI:** 10.3390/sports14050197

**Published:** 2026-05-09

**Authors:** Afroditi Lola, Eleni Bassa, Georgia Stavropoulou, George Giatsis, Konstantinos Chatzinikolaou

**Affiliations:** 1 Laboratory of Motor Behavior and Adapted Physical Activity, School of Physical Education and Sport Sciences, Aristotle University of Thessaloniki, 57001 Thessaloniki, Greece; alola@phed.auth.gr (A.L.); ggiatsis@phed.auth.gr (G.G.); konchatzinikolaou@phed.auth.gr (K.C.); 2 School of Physical Education and Sport Sciences, Aristotle University of Thessaloniki, 62500 Serres, Greece; 3 School of Physical Education and Sport Sciences, Aristotle University of Thessaloniki, 57001 Thessaloniki, Greece

**Keywords:** volleyball, reaction time, perceptual–cognitive abilities, athlete development, sport performance, decision making

## Abstract

Perceptual–cognitive abilities are essential components of performance in volleyball, where players must quickly interpret visual information and respond effectively to rapidly changing game situations. The present study aimed to examine perceptual–cognitive abilities and reaction performance in competitive female volleyball players and to explore how these abilities may contribute to athlete development and training design. Thirty-nine young female volleyball athletes participated in the study and underwent an evaluation of perceptual–cognitive abilities considered critical for volleyball performance. These abilities were assessed through specially designed computer-based tasks delivered via dedicated experimental software, enabling the measurement of reaction time and response accuracy during perceptual–motor processing. Group comparisons did not reveal significant differences between playing positions or competitive levels in the measured perceptual–cognitive abilities. Multivariate and clustering analyses suggested the presence of potential performance patterns characterized by different combinations of reaction speed, response accuracy, and perceptual–cognitive processing. However, these patterns should be interpreted with caution, as the clustering solution showed limited separation (silhouette score = 0.02), indicating an exploratory and non-definitive structure. Overall, the findings highlight the multidimensional nature of perceptual–cognitive performance in volleyball and suggest that athletes may rely on different perceptual–motor strategies when responding to game-related stimuli. From an applied perspective, integrating perceptual–cognitive challenges into training environments may support athlete development and improve decision-making efficiency in dynamic game situations.

## 1. Introduction

Volleyball is a high-speed, open-skill sport that requires athletes to continuously perceive, interpret, and respond to rapidly changing game situations. Due to its fast pace, constant spatial reconfiguration, and the need for rapid decision-making under pressure, the sport places high perceptual–cognitive abilities on players [1]. Successful performance in volleyball depends not only on physical and technical abilities but also on the athlete’s capacity to quickly perceive, process, and respond to information within the game environment. In this context, perceptual–cognitive abilities—such as psychomotor speed, visuospatial working memory, and spatial visualization—are increasingly recognized as important components of sport performance. These abilities enable players to anticipate opponents’ actions, adjust their positioning, and execute tactical decisions within extremely limited timeframes [2,3].

Among these abilities, visual skills play a particularly important role in optimizing performance in team sports such as volleyball, where athletes must continuously interpret visual information and react accordingly. Athletes trained in visually demanding environments demonstrate enhanced dynamic visual acuity, faster reaction time, and improved decision-making accuracy [4,5,6]. In volleyball specifically, athletes with well-developed visual abilities are better able to interpret key cues such as opponent movements, teammate positioning, and the ball’s trajectory, which supports more efficient motor responses and improved task execution during play [7,8]. This capacity to efficiently process visual information is particularly critical in fast-evolving game situations where decisions must be made in fractions of a second.

The perceptual–cognitive abilities of volleyball may vary according to the specific role of the athlete on the court. Different playing positions are likely to involve variations in how athletes interpret and respond to visual and spatial information, although the existing empirical evidence remains limited and not entirely consistent. For example, Outside Hitters must quickly evaluate blocking formations and defensive positioning in order to adapt their attacking strategies during the play. Opposite Hitters frequently participate in complex offensive actions and rely heavily on short-term visuospatial memory and anticipatory processing to coordinate their movements with the setter’s decisions. Similarly, setters must rapidly integrate multiple sources of spatial information to organize offensive plays effectively [7,9,10]. Across all playing positions, the ability to efficiently synchronize visual perception with motor execution is considered a key factor distinguishing high-performing athletes. However, these potential differences should be interpreted with caution, as they have not been consistently demonstrated across studies and may depend on contextual and task-specific factors.

Research has also shown that perceptual–cognitive abilities are closely associated with athletic expertise. Athletes with higher levels of training and competitive experience often demonstrate superior performance in tasks involving visual tracking, prediction, and stimulus discrimination compared with less experienced players [11,12]. These perceptual–cognitive advantages allow elite volleyball players to identify tactical opportunities more effectively and manage the complex spatial and temporal demands of team-based gameplay [13]. Such findings support the view that perceptual–cognitive integration is not only characteristic of high-level performance but may also represent a trainable component of athletic development [5,14].

The development of perceptual–cognitive abilities can be facilitated through structured training environments that expose athletes to sport-specific stimuli and decision-making challenges. Repeated exposure to game-like situations, combined with feedback and deliberate practice, has been shown to enhance attention allocation, cue utilization, and the efficiency of information processing in expert athletes [3]. These adaptations are shaped through both neural and experiential mechanisms, highlighting the importance of integrating perceptual–motor and decision-making demands into sport-specific training environments aimed at long-term athlete development and performance optimization [15,16].

Despite growing interest in perceptual–cognitive performance in sport, research in volleyball has predominantly focused on anthropometric and physical characteristics, such as height, body composition, and morphological features, as key determinants of performance in female players across different playing positions [17]. Comparatively limited attention has been given to the multidimensional assessment of perceptual–cognitive abilities and the use of person-centered analytical approaches to examine how these abilities co-exist within athletes. Therefore, the present study aims to address this gap by combining traditional group comparisons with exploratory cluster analysis to identify latent perceptual–cognitive performance patterns.

The present study adopts an exploratory, hypothesis-generating approach. Therefore, the primary aim of the present study is to identify perceptual–cognitive performance profiles in female volleyball athletes using cluster analysis. Specifically, athletes’ performance was assessed across three core perceptual–cognitive abilities—visuospatial working memory, psychomotor speed, and spatial visualization—using measures of response accuracy (% correct responses) and reaction time (milliseconds). A secondary aim of the study is to explore whether the identified performance profiles are associated with competitive level (Volleyball League and Pre-Volleyball League) and playing position (Outside Hitters and Opposite Hitters). Finally, by identifying perceptual–cognitive performance patterns, this study aims to contribute to a better understanding of cognitive characteristics in volleyball athletes and to provide applied insights for coaches and practitioners regarding training and athlete development.

## 2. Materials and Methods

### 2.1. Participants

The study included a sample of 39 competitive female volleyball athletes competing in the Volleyball League and the Pre-Volleyball League. The Volleyball League represents the highest national competitive level, whereas the Pre-Volleyball League corresponds to a lower, sub-elite division within the Greek volleyball system. The athletes had an average age of 21.59 ± 4.91 years and an average training experience of 11.23 ± 3.49 years. Participants represented two specialized playing positions: Outside Hitters (n = 22) and Opposite Hitters (n = 17). All athletes voluntarily participated in the study and had the right to withdraw at any time. Athletes were excluded if they had experienced a concussion within the past six months, had a history of neurological disorders, presented visual impairments not correctable to normal vision, used medication that could affect cognitive functioning, or failed to complete all perceptual–cognitive assessments. The anonymity of the participants was ensured, and the study was approved by the Research Ethics Committee of the University (Approval No. 148/16.05.2023). A post hoc power analysis was conducted to evaluate the adequacy of the sample size for the MANOVA. With a total sample of 39 athletes, an alpha level of 0.05, and an observed effect size of _p_η^2^ = 0.13, the estimated statistical power was approximately 0.72, indicating moderate statistical power.

### 2.2. Experimental Design

Data collection was conducted in the athletes’ training environment using a laptop computer equipped with E-Prime 3 software and a Chronos response device (Psychology Software Tools, Pittsburgh, PA, USA), which enabled precise measurement of cognitive processing and motor response times. To control for potential confounding factors such as fatigue and learning effects, the order of the cognitive tests was randomized across participants. This procedure minimized potential sequence effects and helped ensure the internal validity of the measurements. The total duration of all the testing sessions was approximately 20 min. The Corsi Block-Tapping Task consisted of fourteen trials, the mental rotation task included twenty-four trials, and the simple reaction time task consisted of eighteen trials. No feedback was provided during testing. The number of trials for each task was selected to ensure a balance between measurement reliability and participant fatigue within a field-based testing context. Specifically, the trial counts were sufficient to obtain stable estimates of performance (reaction time and accuracy), while maintaining a manageable testing duration to preserve participants’ concentration and minimize fatigue-related effects. This approach is consistent with commonly applied protocols in perceptual–cognitive assessment in sport settings.

Short rest intervals were provided between tasks to minimize potential fatigue effects and maintain participants’ concentration throughout the procedure. All testing sessions were conducted under similar conditions, including consistent time-of-day scheduling, to minimize potential variability related to circadian influences on cognitive performance.

### 2.3. Evaluation Tools

A standard laptop computer equipped with E-Prime software and a Chronos keyboard device connected via USB was used to conduct the measurements. E-Prime enables the accurate assessment of perceptual–cognitive abilities such as visuospatial memory, reaction time, and spatial perception, which are considered critical for performance in team sports that require rapid information processing and decision-making.

These perceptual–cognitive abilities were selected due to their relevance to key components of performance in volleyball. Visuospatial working memory supports the temporary storage and updating of spatial information during play, psychomotor speed reflects the efficiency of stimulus–response processing, and spatial visualization contributes to the interpretation of dynamic spatial relationships. Together, these abilities represent core elements of perceptual–motor processing required in such environments. The portable E-Prime setup enabled field-based data collection in the athletes’ training environment, facilitating testing under realistic conditions.

Each participant was seated in front of the computer and completed three computerized tasks using the dedicated Chronos response device. The Chronos system is a multifunctional response and stimulus device designed for use with E-Prime software and provides millisecond-level precision in response time recording. Test instructions were presented in both Greek and English to accommodate the linguistic background of the participants, as the sample included both Greek and international athletes.

### 2.4. Assessment Tests

Three computer-based cognitive tests were administered through the E-Prime software to assess perceptual–cognitive abilities considered important for volleyball performance: visuospatial memory, spatial visualization, and psychomotor speed. These abilities are particularly relevant in volleyball, a fast-paced sport in which athletes must rapidly process visual information, track the movement of the ball and opponents, and respond quickly to changing game situations. In all tests, participants completed three phases: an instruction phase, a short practice phase, and the testing phase. Response accuracy (%) and reaction time (ms) were recorded for each task. Each participant completed a familiarization phase prior to testing to ensure task understanding and reduce potential learning effects.

Visuospatial Memory Assessment. Visuospatial working memory was assessed using the Corsi Block-Tapping Task, a standardized test designed to evaluate the ability to temporarily store and reproduce spatial information. In this task, participants observed sequences of illuminated squares and were required to reproduce them in the correct order. The task started with sequences of two squares, and the sequence length increased when the participant reproduced the sequence correctly. If both sequences at a given level were incorrect, the sequence length decreased. The test consisted of fourteen trials and no feedback was provided during testing. Both response accuracy and reaction time were recorded. Visuospatial memory is particularly important in volleyball, where players must continuously monitor and remember the positions of the ball, teammates, and opponents during rapidly changing game situations.

Spatial Visualization Assessment. Spatial visualization was assessed using a computerized mental rotation task. Participants were shown a letter on the screen and were asked to indicate whether the letter appeared in its normal orientation or as a mirror image. Half of the stimuli were presented normally and the other half as mirrored versions. This task evaluates the ability to mentally process and interpret spatial relationships. In volleyball, spatial visualization helps athletes understand player positioning and anticipate ball trajectories during offensive and defensive actions. Response accuracy and reaction time were recorded for all trials.

Psychomotor Speed Assessment. Psychomotor speed was assessed using a Simple Reaction Time Task developed in E-Prime. During the task, a visual stimulus (a star) appeared at the center of the screen, and participants were instructed to respond as quickly as possible by pressing the “1” key on the Chronos response device. Reaction time was defined as the latency (in ms) between the presentation of the stimulus and the participant’s response. Fast perceptual–motor responses are essential in volleyball, where players must react immediately to ball trajectories and opponent actions. Response accuracy and reaction time were recorded for all trials.

All tasks used in the present study are standardized cognitive assessments widely applied in experimental and sport psychology research, demonstrating acceptable levels of reliability and validity in measuring perceptual–cognitive performance.

### 2.5. Statistical Analysis

Descriptive statistics were initially calculated for the anthropometric characteristics of the participants. Means, standard deviations, medians, range, variance, minimum, and maximum values were reported to provide a comprehensive description of the sample. To examine potential differences between high-performance athletes (Volleyball League) and sub-elite athletes (Pre-Volleyball League) in the three perceptual–cognitive abilities (visuospatial memory, psychomotor speed, and spatial visualization), independent samples *t*-tests were conducted for both reaction time and response accuracy. A multivariate analysis of variance (MANOVA) was performed to investigate whether athletes’ playing positions (Outside Hitters and Opposite Hitters) were associated with differences in performance across the perceptual–cognitive tests. Prior to conducting inferential analyses, statistical assumptions were examined. Normality of the data was assessed using the Shapiro–Wilk test, as well as inspection of Q–Q plots and skewness–kurtosis values. Homogeneity of variances for *t*-tests was evaluated using Levene’s test. For the MANOVA, the assumption of equality of variance–covariance matrices was assessed using Box’s M test. In addition, multicollinearity among dependent variables was checked using correlation matrices. All assumptions were deemed acceptable for the analyses conducted. Furthermore, K-means cluster analysis was applied to classify athletes based on their performance in the three perceptual–cognitive abilities. Subsequently, a one-way analysis of variance (ANOVA) was conducted to examine differences between the identified clusters. Given the multidimensional nature of the variables, multiple statistical approaches were applied to capture different aspects of performance. However, due to the exploratory nature of the study and the limited sample size, results should be interpreted cautiously. Outliers in reaction time data were examined and handled using standard criteria. Specifically, extreme values exceeding ±3 standard deviations from the mean were identified and excluded from the analysis to reduce the influence of atypical responses.

## 3. Results

The results obtained were analyzed using descriptive statistics to highlight the parameters of the anthropometric characteristics. The following data are presented: means, medians, prevalent values, standard deviations, range, variance, minimum, maximum, and sum. Table 1 presents the descriptive statistics for the variables of age, height, weight, and total years of involvement. The age of the athletes is presented with a minimum value of 13 and a maximum value of 34 (M = 21.59, sd = 4.91). Height is also shown with a minimum value of 1.45 cm and a maximum value of 1.95 cm (M = 1.87, sd = 0.09). Weight is following with minimum value 48 and maximum value 90 (M = 67.53, sd = 9.07). Years of training present a minimum value of 8 and a maximum value of 18 (M = 11.23, sd = 3.49).

Furthermore, descriptive analysis revealed that the majority of players (22) played as Outside Hitters, followed by 17 Opposite Hitters. The majority of participants (20) were affiliated with the Volleyball League, while 19 were from the Pre-Volleyball League. Concerning hand dominance, 35 players were classified as right-handed, while 4 were identified as left-handed. The study revealed a comparable trend in the prevalence of ocular dominance, with 30 subjects demonstrating right-eye dominance and 9 exhibiting left-eye dominance.

To investigate whether there were statistically significant differences between the high-performance athletes and sub-elite athletes and the total years in the three perceptual–cognitive abilities in terms of reaction time and accuracy of response, an independent T-test was used. No statistically significant differences were observed between groups across all perceptual–cognitive measures. For playing position, visuospatial memory performance in the Corsi Tapping Block test showed no differences in accuracy, t(37) = 0.35, *p* = 0.73, Cohen’s d = 0.11, 95% CI [−0.52, 0.75], or reaction time, t(37) = −1.21, *p* = 0.23, Cohen’s d = −0.39, 95% CI [−1.03, 0.25]. Psychomotor speed (Simple Reaction Time test) also showed no differences in reaction time, t(37) = −0.88, *p* = 0.38, Cohen’s d = −0.29, 95% CI [−0.92, 0.35]. Similarly, spatial visualization (Rotations of Mental Images test) showed no differences in accuracy, t(37) = 0.44, *p* = 0.66, Cohen’s d = 0.14, 95% CI [−0.49, 0.77], or reaction time, t(37) = 1.75, *p* = 0.09, Cohen’s d = 0.57, 95% CI [−0.08, 1.21]. For expertise level, visuospatial memory (Corsi Tapping Block test) showed no differences in accuracy, t(37) = −1.14, *p* = 0.26, Cohen’s d = −0.37, 95% CI [−1.00, 0.27], or reaction time, t(37) = −0.83, *p* = 0.41, Cohen’s d = −0.27, 95% CI [−0.90, 0.37]. Psychomotor speed (Simple Reaction Time test) showed no differences in reaction time, t(37) = −1.38, *p* = 0.18, Cohen’s d = −0.44, 95% CI [−1.07, 0.20], while spatial visualization (Rotations of Mental Images test) showed no differences in accuracy, t(37) = −0.53, *p* = 0.60, Cohen’s d = −0.17, 95% CI [−0.80, 0.46], or reaction time, t(37) = −0.49, *p* = 0.63, Cohen’s d = −0.16, 95% CI [−0.79, 0.47].

**Figure 1:**
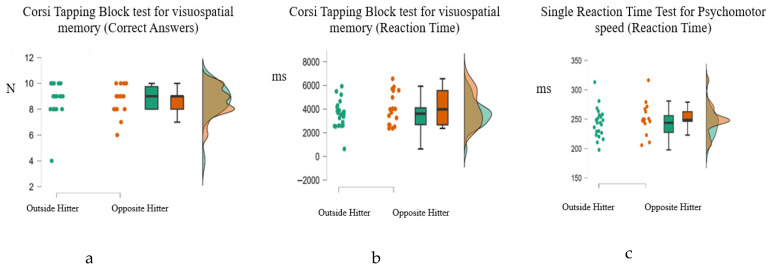
Raincloud plots illustrating the distribution of athletes’ performance by playing position for (**a**) Corsi Block-Tapping Test (visuospatial memory accuracy), (**b**) Corsi Block-Tapping Test (visuospatial memory reaction time), and (**c**) Simple Reaction Time Test (psychomotor speed reaction time). The plots combine raw data points, boxplots, and density distributions to provide a comprehensive visualization of group differences and variability within each condition.

**Figure 2:**
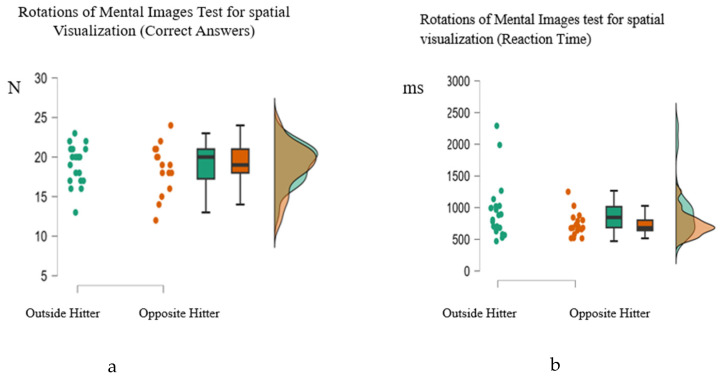
Raincloud plots illustrating the distribution of athletes’ performance by playing position for (**a**) Rotations of Mental Images Test (spatial visualization accuracy: correct responses), and (**b**) Rotations of Mental Images Test (spatial visualization reaction time). The plots integrate raw data points, boxplots, and density distributions to provide a comprehensive visualization of central tendency, variability, and distributional overlap between groups.

Descriptive statistics indicated minor differences between playing positions. In the visuospatial memory test, Opposite Hitters exhibited a slightly lower mean score in finding the correct answers (M = 8.59, sd = 1.21) than the Outside Hitters (M = 8.73, sd = 1.32. Concerning the reaction time in the visuospatial memory test, Outside Hitters demonstrated a fast reaction time (M = 3579.51, sd = 1163.53), while Opposite Hitters exhibited a slower reaction time (M = 4074.85 ms, sd = 1389.57). In the psychomotor speed test, all groups demonstrated comparable performance in finding the correct answers, with Outside Hitters achieving a mean score (M = 17.95, sd = 0.21) and Opposite Hitters attaining perfect scores (M = 18, sd = 0.00). The mean reaction times in this test were fast (M = 243.66, sd = 25.37) for Outside Hitters, and slower (M = 250.94, sd = 25.64) for Opposite Hitters. In the spatial visualization test, Opposite Hitters scored the highest score in finding the correct answers (M = 18.71, sd = 3.06), followed by Outside Hitters (M = 19.09, SD = 2.43). Finally, the reaction times in the spatial visualization test showed that Outside Hitters had a mean score (M = 936.79, sd= 443.71), while Opposite Hitters responded faster (M = 734.82, sd = 190.82). However, these differences were small and not statistically significant. This information is presented in Table 2 and Figure 1 and Figure 2. Manova was used to identify if there were statistically significant differences among the athletes’ performance concerning their position. However, no statistically significant multivariate effect of position was observed, Wilks’ Λ = 0.87, F(6, 32) = 0.77, *p* = 0.602, pη^2^ = 0.13.

**Figure 3:**
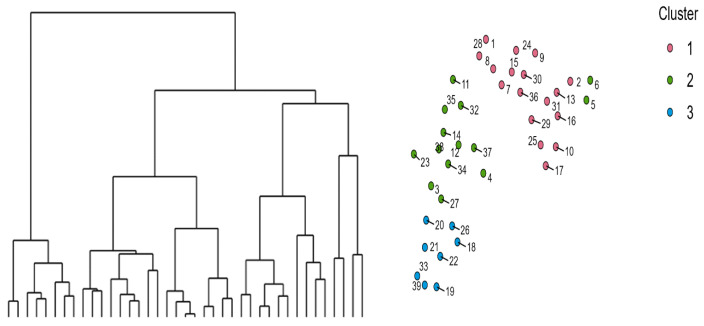
Differences between the three clusters concerning the athletes’ performance in the Corsi Tapping Block test for visuospatial memory, the Single Reaction Time test for psychomotor speed, the Rotations of Mental Images test for spatial visualization, and Expertise.

K-means cluster analysis was then used to categorize the athletes into groups based on their performance in the skills: visuospatial memory, psychomotor speed, and spatial visualization. The results indicated that a three-cluster solution was selected for exploratory interpretation based on evaluation of cluster centroids, within-cluster sum of squares, and analysis of variance. Convergence was achieved, indicating convergence of the algorithm. Initial cluster separation (smallest initial distance = 5.29) allowed convergence of the algorithm, yielding a low silhouette score (0.02), indicating limited separation between clusters; therefore, results should be interpreted cautiously as exploratory. Cluster centroids indicated that Cluster 1 (moderate accuracy, moderate reaction time, moderate expertise) (n = 16) was characterized by medium accuracy in Corsi Tapping Block test for visuospatial memory, Slow Reaction time in Single Reaction Time Test for psychomotor speed, low accuracy in Rotations of Mental Images Test for spatial visualization, fast reaction time in Rotations of Mental Images Test for spatial visualization, and medium expertise. Cluster 2 (lower accuracy, faster reaction time, higher expertise) (n = 9) showed low accuracy in the Corsi Tapping Block test for visuospatial memory, fast Reaction Time in the Single Reaction Time Test for psychomotor speed, moderate accuracy and reaction time in the Rotations of Mental Images Test for spatial visualization, and high expertise. Cluster 3 (higher accuracy, moderate-to-slower reaction time, lower expertise) (n = 14) had high accuracy in the Corsi Tapping Block test for visuospatial memory and the Rotations of Mental Images Test for spatial visualization, medium reaction time in Single Reaction Time Test for psychomotor speed, slow reaction time in Rotations of Mental Images Test for spatial visualization, and low expertise. Table 3 shows these characteristics, and Figure 3 presents the qualitative characteristics.

An analysis of variance was conducted in order to identify how the three clusters were formulated. The expertise contributed significantly to the clusters’ formulation, with F(2) = 18.73, *p* < 0.001.

### Rotations of Mental Images Test for Spatial Visualization

(Correct Answers) also contributed significantly to the clusters’ formulation, with F(2) = 12.57, *p* < 0.001. Single Reaction Time test for psychomotor speed (Reaction Time) followed which also contributed significantly, with F(2) = 8.31, *p* < 0.01. With less contribution Corsi Tapping Block test for visuospatial memory (Correct Answers) and Rotations of Mental Images test for spatial visualization (Reaction Time) followed, with F(2) = 6.12, *p* < 0.01 and F(2) = 6.51, *p* < 0.01, respectively.

## 4. Discussion

The present study examined perceptual–cognitive abilities in competitive female volleyball players by analyzing visuospatial memory, psychomotor speed, and spatial visualization in relation to playing position and competitive level. The findings of the present study should be interpreted within an exploratory, hypothesis-generating framework. While no statistically significant differences were found between athletes competing in the Volleyball League and those in the Pre-Volleyball League, or between Outside Hitters and Opposite Hitters, the cluster analysis revealed potential cognitive performance patterns among the athletes. These findings may suggest that perceptual–cognitive performance in volleyball may be better understood through individual patterns of performance rather than through traditional categorizations based solely on playing position or competition level.

More specifically, the cluster analysis identified three potential different cognitive profiles (patterns) characterized by distinct combinations of reaction speed, response accuracy, and expertise. However, given the small sample size and low cluster separation, these patterns should be interpreted cautiously as exploratory and not as definitive classifications. These results are broadly consistent with contemporary perspectives in sport science suggesting that perceptual–cognitive expertise develops primarily through accumulated experience and training exposure rather than being strictly determined by playing position [3,18]. In fast-paced sports such as volleyball, where athletes must constantly process visual information and react to rapidly changing game situations, the ability to efficiently integrate perception and action represents an essential component of performance.

The identification of three cognitive profiles also highlights the multidimensional nature of perceptual–cognitive performance. Athletes with greater expertise tended to demonstrate faster reaction times, even when this was accompanied by moderate levels of response accuracy. This pattern may reflect the development of automaticity in decision-making processes, which is considered a hallmark of expert performance [1,16]. Experienced athletes often rely on proceduralized cognitive schemas that allow them to respond rapidly to environmental stimuli under time constraints.

At the same time, athletes with lower expertise demonstrated higher response accuracy but slower reaction times, indicating a more deliberate information-processing strategy. This pattern is consistent with the well-established speed–accuracy trade-off, according to which faster responses are often associated with reduced accuracy, particularly in high-pressure environments that require rapid decisions [1]. From a performance perspective, faster reaction times are functionally linked to more efficient perception–action coupling and the ability to execute motor responses under time constraints, which is critical in dynamic sport environments such as volleyball. Previous research has shown that athletes with faster perceptual–motor processing are better able to respond to rapidly changing stimuli, even when this involves a trade-off with response accuracy. In competitive sport settings such as volleyball, athletes frequently prioritize speed of response over absolute precision in order to maintain the tempo of play and respond effectively to dynamic situations.

Recent evidence further supports this relationship, demonstrating strong associations between reaction time and agility performance when assessed through perception–action technological devices. For example, Mancini et al. [19] reported very high correlations between reaction time and agility in young athletes, suggesting that faster perceptual–motor responses may play a predictive role in sport performance. These findings reinforce the ecological relevance of reaction time as a key component of performance in dynamic, open-skill sports such as volleyball.

Interestingly, no statistically significant differences were observed between playing positions, which contrasts with some previous research suggesting position-specific perceptual–cognitive demands [2,20]. Although descriptive statistics indicated minor variations between groups, these differences were not statistically significant and therefore should not be interpreted as evidence of functional or position-specific adaptations. Instead, the present findings suggest that perceptual–cognitive performance may not be strongly determined by playing position, at least within the context of the tasks and sample examined in this study.

From a training perspective, these findings highlight the importance of integrating perceptual–cognitive elements into volleyball training environments. Volleyball requires athletes to rapidly interpret visual information, anticipate opponents’ actions, and execute motor responses under time pressure. Therefore, training programs that incorporate perceptual–motor challenges and sport-specific decision-making tasks may contribute to the development of these abilities and ultimately may support performance.

Previous studies support this applied perspective. Mancini et al. [19] reported that the integration of light-based perception–action training devices into volleyball training programs resulted in improvements in movement speed and reaction time. Similarly, Loureiro et al. [21] emphasized the importance of perceptual–cognitive abilities for offensive decision-making in volleyball, suggesting that training environments should incorporate contextualized game situations to enhance decision-making processes. Alves et al. [22] also demonstrated that elite volleyball players exhibit greater perceptual and tactical efficiency during game transitions, highlighting the importance of perceptual–cognitive development for performance under dynamic conditions.

The present study contributes to this growing body of literature by showing that athletes may be grouped according to perceptual–cognitive performance profiles rather than strictly according to their playing position. This finding suggests that individualized cognitive profiling may represent a potentially useful approach for optimizing training design and athlete development. Instead of assuming that cognitive demands are determined solely by position, coaches and practitioners may benefit from assessing athletes’ perceptual–cognitive strengths and tailoring training stimuli accordingly.

Another important methodological contribution of the study is the use of field-based cognitive assessments administered through E-Prime software. Conducting the tests within the athletes’ training environment may contribute to the ecological validity of the measurements and allow perceptual–cognitive performance to be assessed under conditions closer to real sport contexts [15]. However, it is important to note that ecological validity depends not only on the testing environment but primarily on the representativeness of the tasks. In this respect, the tasks used in the present study do not fully capture key features of sport-specific performance, such as anticipation, real-time decision-making, and dynamic interaction. The use of the Chronos response device also ensured millisecond-level precision in the measurement of reaction times, which is essential when evaluating rapid perceptual–motor responses.

The use of cluster analysis represents an additional contribution of the present study. Traditional between-group comparisons often assume homogeneity within groups, whereas cluster analysis allows the identification of naturally occurring subgroups within the sample. This person-centered analytical approach provides a deeper understanding of variability among athletes and may offer exploratory insights for individualized training and talent development strategies.

The absence of statistically significant differences between positions and levels should be interpreted with caution. While this finding may reflect a true lack of differences between groups, alternative explanations should also be considered. In particular, the sensitivity and specificity of the measurement tools may have been insufficient to detect subtle variations. Furthermore, methodological factors such as sample size and statistical power may have limited the ability to identify small or moderate effects. Therefore, the possibility that meaningful differences exist but were not detected cannot be excluded.

Overall, the findings highlight the complexity of perceptual–cognitive abilities development in team sports and emphasize the importance of multidimensional assessment approaches. While playing position alone may not fully determine cognitive performance characteristics, the integration of perceptual–cognitive training within long-term athlete development programs may play a supportive role in optimizing volleyball performance.

However, it is important to acknowledge several methodological limitations that should be considered when interpreting these findings. A key limitation of the present study is the relatively small sample size (n = 39), which becomes particularly relevant when participants are divided into subgroups based on competitive level and playing position. As a result, some subgroup analyses are based on very small cell sizes, which may reduce statistical power and increase the risk of unstable estimates. Therefore, findings from these comparisons should be interpreted with caution and considered exploratory in nature. In addition, the relatively small cluster sizes and the limited overall sample may affect the stability and generalizability of the clustering solution. More robust validation procedures, such as bootstrap resampling, comparison of alternative cluster solutions, or hierarchical clustering methods, were not applied in the present study and should be considered in future research. Therefore, the identified clusters should be interpreted as exploratory patterns rather than stable or generalizable classifications. Another limitation of the present study is the absence of statistically significant differences between conditions. This may be partly explained by suboptimal statistical power, potentially due to the sample size, which may have limited the ability to detect small or moderate effects. Therefore, the possibility of a Type II error cannot be excluded. Additionally, the ecological validity of the tasks should be considered. It is also important to distinguish between ecological validity in terms of the testing environment and the representativeness of the tasks. Although the assessments were conducted in a field-based setting, the tasks used in this study primarily measure general perceptual–cognitive functions and do not fully replicate the anticipatory, interactive, and decision-making demands of real volleyball performance. Therefore, the findings should be interpreted as reflecting general cognitive processing capacities that may contribute to sport performance, rather than as direct measures of sport-specific behavior. Although the measures were administered under controlled conditions to ensure experimental consistency, such settings may not fully capture the complexity of real-world environments. As a result, participants’ performance in the study may not entirely reflect their behavior in everyday contexts, which may limit the generalizability of the findings. Future research using more naturalistic designs would help to address this limitation.

## 5. Conclusions

The present study examined perceptual–cognitive abilities in competitive female volleyball players by analyzing visuospatial memory, psychomotor speed, and spatial visualization in relation to playing position and competitive level. The findings indicated that these cognitive abilities did not differ significantly between athletes competing at different performance levels or between Outside Hitters and Opposite Hitters.

However, the cluster analysis suggested the presence of potential cognitive performance profiles among the athletes, which may indicate that perceptual–cognitive performance in volleyball could be better understood through individual performance patterns rather than traditional categorizations based solely on playing position. Nevertheless, these findings should be interpreted with caution, as the clustering results are exploratory in nature and showed limited separation, and therefore do not represent definitive classifications.

Within this exploratory context, the results may provide preliminary support for the multidimensional nature of perceptual–cognitive performance in volleyball. Athletes may rely on different cognitive strategies, balancing reaction speed and response accuracy depending on their level of expertise and perceptual–motor processing style. This observation supports the view that perceptual–cognitive abilities represent a trainable component of performance and may develop differently across athletes.

From an applied perspective, these findings should be interpreted with caution and may provide only preliminary insights into how perceptual–cognitive characteristics could be considered in athlete development and training design. Rather than assuming uniform cognitive demands based on playing position, coaches and practitioners may benefit from assessing athletes’ perceptual–cognitive characteristics and tailoring training environments accordingly.

Overall, the findings emphasize the importance of integrating perceptual–cognitive assessment and training into volleyball development programs. Such approaches may support more effective decision-making, faster perceptual–motor responses, and improved performance in dynamic game situations, although further research using larger samples and more sport-specific assessment tools is needed to confirm these relationships.

## 6. Suggestions for Practical Application

The findings of the present study provide several practical implications for coaches and performance specialists working in volleyball. First, perceptual–cognitive abilities such as visuospatial memory, psychomotor speed, and spatial visualization should be considered important components of athlete development programs. Integrating perceptual–motor challenges into regular training sessions may improve athletes’ ability to process visual information and respond effectively to rapidly changing game situations.

Second, coaches should consider incorporating perceptual–cognitive training tasks that simulate real game conditions, such as drills requiring rapid decision-making, visual tracking, and anticipation of opponents’ actions. These types of training activities can enhance athletes’ perceptual–cognitive processing and reaction speed during competitive play.

Third, the identification of different cognitive performance profiles suggests that athletes may benefit from individualized training approaches. Assessing perceptual–cognitive characteristics may allow coaches to tailor training stimuli according to each athlete’s strengths and weaknesses, rather than relying solely on position-based training methods.

Finally, the integration of field-based cognitive assessment tools, such as computerized perceptual–cognitive tests, may help coaches monitor athletes’ cognitive performance and evaluate the effectiveness of training interventions over time.

## Figures and Tables

**Table 1:** Descriptive data about anthropometric characteristics of age, height, weight, and years of training.

	Age	Height	Weight	Years of Training
N	39	39	39	39
Mean ± S.D.	21.59 ± 4.91	1.87 ± 0.09	67.53 ± 9.07	11.23 ± 3.49
Median	20	1.85	68	10
Mode	19	1.80	70	8
Variance	24.14	0.01	82.2	12.18
Range	21	0.50	42	10
Minimum	13	1.45	48	8
Maximum	34	1.95	90	18

**Table 2:** Means and Standard deviations in memory, single reaction time, and perception test concerning the athletes’ position.

Tests	Outside Hitter	%	Opposite Hitter	%
Corsi Tapping Block test for visuospatial memory (Correct Answers)	8.73 ± 1.32	87.3%	8.59 ± 1.21	85.9%
Corsi Tapping Block test for visuospatial memory (Reaction Time)	3579.51 ± 1163.53 (ms)		4074.85 ± 1389.57 (ms)	
Single Reaction Time Test for psychomotor speed (Correct Answers)	17.95 ± 0.21	99.72%	18 ± 0.00	100%
Single Reaction Time test for psychomotor speed (Reaction Time)	243.66 ± 25.37 (ms)		250.94 ± 25.64 (ms)	
Rotations of Mental Images Test for spatial visualization (Correct Answers)	19.09 ± 2.43	79.54%	18.71 ± 3.06	77.96%
Rotations of Mental Images test for spatial visualization (Reaction Time)	936.79 ± 443.71 (ms)		734.88 ± 190.82 (ms)	

**Table 3:** Qualitative description of the three clusters.

Variables	Cluster 1 (Moderate Accuracy, Moderate Reaction Time, Moderate Expertise)	Cluster 2 (Lower Accuracy, Faster Reaction Time, Higher Expertise)	Cluster 3 (Higher Accuracy, Moderate-to-Slower Reaction Time, Lower Expertise)
Corsi Tapping Block test for visuospatial memory (Correct Answers)	Medium Accuracy	Lower accuracy	Higher accuracy
Single Reaction Time test for psychomotor speed (Reaction Time)	Slower Reaction Time	Faster Reaction Time	Medium Reaction Time
Rotations of Mental Images Test for spatial visualization (Correct Answers)	Lower Accuracy	Medium accuracy	Higher accuracy
Rotations of Mental Images test for spatial visualization (Reaction Time)	Faster Reaction Time	Medium Reaction Time	Slower Reaction Time
Expertise	Medium Expertise	Higher Expertise	Lower Expertise

## Data Availability

Data are available upon request due to ethical restrictions.
